# Supplementary material for: Pan-Renal Cell Carcinoma classification and survival prediction from histopathology images using deep learning
Source: Sci Rep. 2019 Jul 19;9:10509. doi: 10.1038/s41598-019-46718-3 (PMC6642160; doi:10.1038/s41598-019-46718-3)
Supplement: Supplementary file 1 — Supplementary Information [file 41598_2019_46718_MOESM1_ESM.pdf]

Supplementary Information

Pan-Renal Cell Carcinoma classification and survival prediction from histopathology images using deep learning

Sairam Tabibu[1,*], P.K. Vinod[2,+] and C.V. Jawahar[1,†]

[1]Center for Visual Information Technology, IIIT, Hyderabad, India
[2]Center for Computational Natural Sciences and Bioinformatics, IIIT Hyderabad, Hyderabad, India
[*]t.sairam.ece13@iitbhu.ac.in
[+]vinod.pk@iiit.ac.in
[†]jawahar@iiit.ac.in

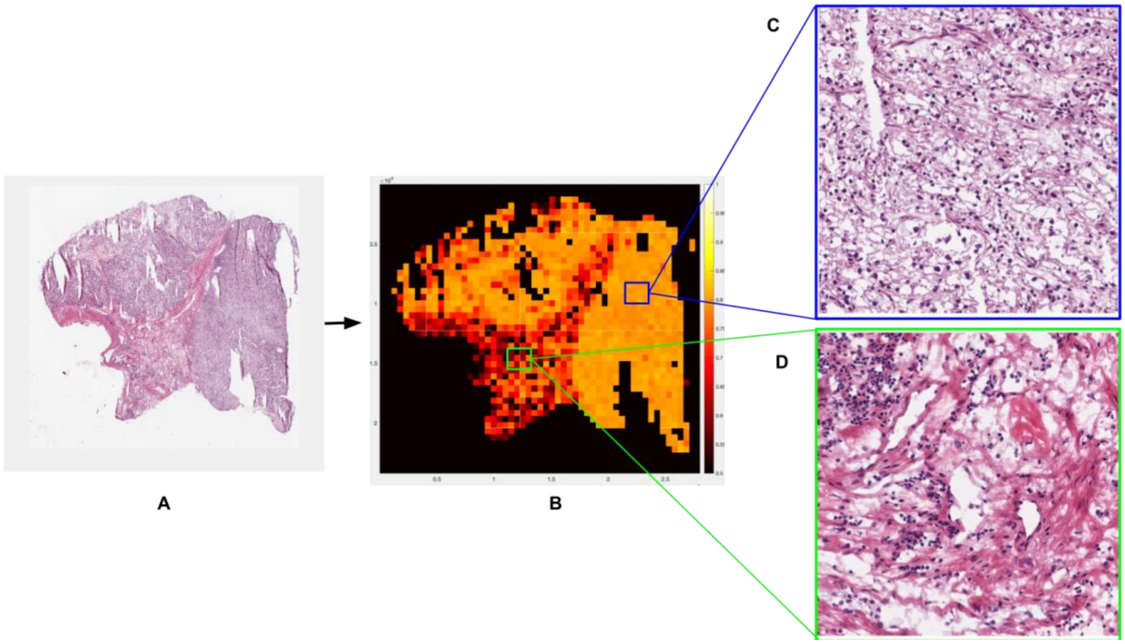

Supplementary Figure 1: Visualization of High probability regions of KIRC. High probability regions (displayed in blue) and low probability regions (displayed in green).

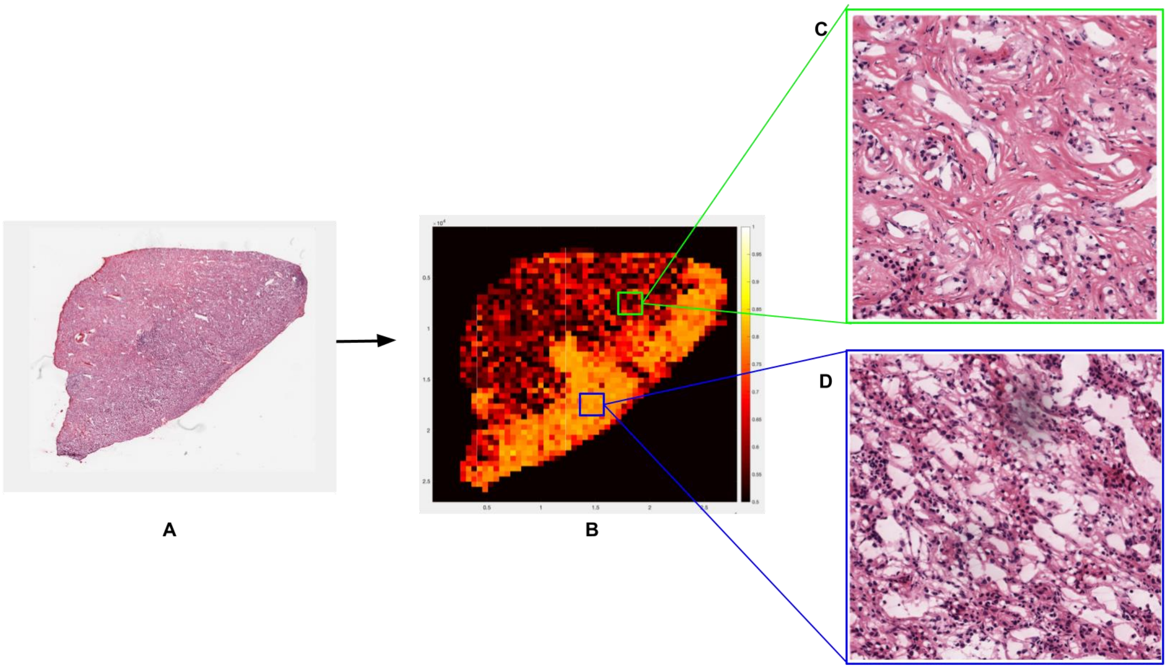

Supplementary Figure 2: Visualization of high probability regions of KIRC. High probability regions (shown in blue) and low probability regions (shown in green).

Supplementary Table 1: Number of slides and patches of normal and tumor at different resolutions for KIRC.

Resolution	Tissue	Training	Validation	Testing
20X	Cancerous	719(213302)	154(55463)	154(42907)
20X	Normal	379(115856)	81(23434)	81(23401)
40X	Cancerous	719(672586)	154(163093)	154(130399)
40X	Normal	379(428719)	81(88406)	81(92581)

Supplementary Table 2: Number of slides and patches of normal and tumor at different resolutions for KIRP.

Resolution	Tissue	Training	Validation	Testing
20X	Cancerous	179(74626)	39(23613)	38(13577)
20X	Normal	33(6102)	7(1285)	7(908)
40X	Cancerous	179(235858)	39(83855)	38(81267)
40X	Normal	33(21614)	7(4365)	7(5418)

Supplementary Table 3: Number of slides and patches of normal and tumor at different resolutions for KICH.

Resolution	Tissue	Training	Validation	Testing
20X	Cancerous	106(202912)	23(50811)	22(45218)
20X	Normal	58(97899)	13(20610)	12(32538)
40X	Cancerous	106(647960)	23(172033)	22(164026)
40X	Normal	58(374812)	13(78663)	12(126294)

Supplementary Table 4: Model performance for each subtype with Vanilla CNN in the subtype classification.

Model	Subtype	Accuracy
Resnet 18	KIRC	97.09%
	KIRP	69.75%
	KICH	85.36%
Resnet 34	KIRC	97.35%
	KIRP	72.49%
	KICH	79.72%

Supplementary Table 5: Model performance for each subtype with DAG-SVM + Vanilla CNN in the subtype classification.

Model	Subtype	Accuracy
Resnet 18	KIRC	94.84%
	KIRP	77.25%
	KICH	95.12%
Resnet 34	KIRC	91.30%
	KIRP	82.41%
	KICH	95.47%

Supplementary Table 6: Effect of data augmentation and weighted sampling on subtype classification.

**Model**	**Accuracy**	**Precision**	**Recall**	**Kappa score**
Resnet 18	87.69	88.82	83.66	0.794
Resnet 34	86.19	88.30	83.18	0.770
Resnet 18(DAG-SVM)	**92.62**	90.78	89.07	**0.877**
Resnet 34(DAG-SVM)	91.96	88.94	87.92	0.867
Resnet 18(weighted sampling)	91.44	89.37	89.77	0.859
Resnet 34(weighted sampling)	92.16	90.31	90.76	0.871
Resnet 18(weighted sampling + DAG-SVM)	**93.79**	92.40	90.48	**0.897**
Resnet 34(weighted sampling + DAG-SVM)	**94.07**	90.85	92.67	**0.902**
Resnet 18 (augmentation)	91.40	90.77	87.86	0.857
Resnet 34 (augmentation)	91.47	90.38	89.44	0.859
Resnet 18 (augmentation + DAG-SVM)	**93.68**	91.43	90.86	**0.895**

Supplementary Table 7: Classification of normal tissues of RCC subtypes using the model trained to detect subtypes.

**Model**	**Precision**	**Recall**	**AUC**
Resnet 18	79.88%	79.85%	0.85
Resnet 34	79.44%	79.12%	0.85

Supplementary Table 8: KIRC Patient characteristics.

KIRC	Summary	Median
**Number of Patients**	469	
**Status**		
Alive	310	
Dead	159	
**Age**	26-90 years	61.42 years
**Gender**		
Male	300	
Female	169	
**Grade**		
1	10	
2	188	
3	175	
4	58	
**Stage**		
1	228	
2	49	
3	117	
4	73	

Supplementary Table 9: Features extracted for survival analysis.

Tumor Shape Features	Tumor Nuclei Features
Main region area	Total Area
Main region convex area	Total filled area
Main region filled area	Total convex area
Main region perimeter	Total Perimeter
Main region major axis	Total Minor axis
Total peri(squared) by area	Total Major Axis
Main region minor axis	Total peri(squared) by area
Main region peri(squared) by area	
Main region eccentricity	
Main region solidity	
Main region angle	
Total area	
Total convex area	
Total filled area	
Total major axis	
Total minor axis	
Total perimeter	
Total peri(squared) by area	
